# Association Between Lipid Metabolism and Obesity

**DOI:** 10.3390/nu18121963

**Published:** 2026-06-18

**Authors:** Renalison Farias-Pereira, Keciany Alves de Oliveira

**Affiliations:** 1 Department of Biological Sciences, The Dorothy and George Hennings College of Science, Mathematics and Technology, Kean University, Union, NJ 07083, USA; 2 Higher Institute of Biomedical Sciences, State University of Ceara-UECE, Fortaleza 60714-903, CE, Brazil; keciany.oliveira@uece.br; 3 Postgraduate Program in Nutrition and Health, State University of Ceara-UECE, Fortaleza 60714-903, CE, Brazil

This Special Issue on the “Association Between Lipid Metabolism and Obesity” includes studies that focus on lipid metabolic pathways and their relationship with obesity. The bidirectional relationship between unbalanced lipid metabolism and diet-related diseases, such as obesity, is an intricate and complex association that has been explored in multiple ways to prevent and treat the obesity epidemic [1]. While increased lipogenesis is a recognized hallmark of fat accumulation, the specific roles of fatty acid β-oxidation, lipolysis, and lipid transport remain areas of intense investigation [2,3,4]. Therefore, further studies are still needed to elucidate the biological roles of molecular targets in lipid metabolism and to determine, for example, whether targeting them could contribute to or prevent excessive fat accumulation. The five articles featured in this issue offer different kinds of evidence on the dynamics linking dietary components and metabolism. These studies use human clinical data, rodent models, and alternative biological models to identify novel therapeutic molecular targets and bioactive interventions.

One of the focuses showcased in this Special Issue is the investigation of natural-derived extracts and dietary components in modulating lipid homeostasis. A comprehensive systematic review of yacon syrup (*Smallanthus sonchifolius*) highlights its potential role as a functional supplement against increased body weight and waist circumference. Contribution 1’s analyses revealed that overall, the high concentration of fructooligosaccharides (FOS) in yacon syrup may selectively stimulate beneficial gut bacteria, leading to the production of short-chain fatty acids (SCFAs) that improve insulin sensitivity, reduce LDL cholesterol, and enhance satiety via hormones like GLP-1. However, in the reviewed studies, yacon syrup’s effects were found to be dependent on dose, duration, and population.

Contribution 2 explored the metabolic benefits of hexanoic acid, a medium-chain fatty acid (MCFA) found in milk and coconut oil. Their study demonstrates that hexanoic acid supplementation in mice fed a high-fat diet (HFD) improved hyperglycemia and hyperinsulinemia while suppressing adipose tissue accumulation. Hexanoic acid reduced the transcript levels of carbohydrate response element-binding protein (*Chrebp*) and fatty acid synthase (*Fasn*), both related to fatty acid biosynthesis, in epididymal adipose tissues. Since lipid metabolism is also associated with overall energy homeostasis, this study showed that hexanoic acid appears to be more potent than butyric acid in maintaining glucose homeostasis, potentially by restoring the expression of genes associated with hepatic gluconeogenesis and increasing plasma GLP-1 levels.

The interaction of hexaraphane, an isothiocyanate derived from wasabi, with the peroxisome proliferator-activated receptor α (PPARα) signaling pathway was investigated in Contribution 3. Their findings suggest that hexaraphane acts as a PPARα activator, upregulating enzymes like carnitine palmitoyltransferase 1A(CPT1A) to promote fatty acid β-oxidation. Hexaraphane increased the hepatic senescence marker sirtuin 1 (Sirt1) expression, which was negatively correlated with plasma triglyceride levels in aged mice. These effects suggested that hexaraphane has the potential to reduce age-related weight gain and plasma triglyceride levels.

To understand the molecular pathways of bioactive compounds on lipid metabolism, researchers often turn to alternative biological models. Contribution 4 used the invertebrate model *Caenorhabditis elegans* to study the anti-obesity effects of esculetin, a coumarin found in herbal plants. Esculetin reduced fat accumulation through mechanisms dependent on the insulin signaling and AMP-activated protein kinase (AMPK) pathways. By upregulating genes such as *atgl-1*, esculetin likely enhanced lipolysis, offering a potential strategy for pharmacological intervention in lipid metabolism disorders. On the other hand, the fat-lowering effects of esculetin were not dependent on molecular targets related to lipogenesis or fatty acid β-oxidation-related molecular targets, such as sterol regulatory element binding protein (SREBP) and PPARα.

The nuances of lipid transport and the organ-specific contributions of a liver fatty acid-binding protein (LFABP) to metabolic health were further elucidated in Contribution 5. This study uses tissue-specific LFABP knockout mice. The ablation of LFABP either in liver or intestine leads to increased weight gain and fat mass, without changes in glucose homeostasis. The increased weight was associated with lower fatty acid uptake capacity in liver or intestine. While liver-specific ablation had little effect on intestinal lipid metabolic genes, intestine-specific ablation led to changes in the expression of hepatic genes involved in fatty acid synthesis (e.g., *Fasn*) and oxidation (e.g., *Cpt1a*), suggesting important inter-organ crosstalk.

In conclusion, these studies advance our understanding of how dietary interventions and molecular signaling pathways regulate body fat and lipid metabolism. From the gut–microbiota axis modulated by yacon syrup to the PPARα-mediated longevity benefits of hexaraphane, these articles suggest that targeting specific lipid metabolism pathways may prevent and/or treat obesity-associated complications. We hope this Special Issue serves as a valuable resource for researchers trying to develop functional foods or therapeutic molecular strategies to combat the global obesity epidemic.

## References

[1] Nussbaumerova B., Rosolova H. (2023). Obesity and Dyslipidemia. Curr. Atheroscler. Rep..

[2] An S.M., Cho S.H., Yoon J.C. (2023). Adipose Tissue and Metabolic Health. Diabetes Metab. J..

[3] Mota de Sá P., Richard A.J., Hang H., Stephens J.M. (2017). Transcriptional Regulation of Adipogenesis. Compr. Physiol..

[4] Proença A.R., Sertié R.A., Oliveira A.C., Campaña A.B., Caminhotto R.O., Chimin P., Lima F.B. (2014). New Concepts in White Adipose Tissue Physiology. Braz. J. Med. Biol. Res..

